# Identification of the Binding Epitope of an Anti-Mouse CCR6 Monoclonal Antibody (C_6_Mab-13) Using 1× Alanine Scanning

**DOI:** 10.3390/antib12020032

**Published:** 2023-04-28

**Authors:** Tomohiro Tanaka, Mayuki Tawara, Hiroyuki Suzuki, Mika K. Kaneko, Yukinari Kato

**Affiliations:** 1 Department of Molecular Pharmacology, Tohoku University Graduate School of Medicine, 2-1 Seiryo-machi, Aoba-ku, Sendai 980-8575, Japan; tomohiro.tanaka.b5@tohoku.ac.jp (T.T.); tawara.mayuki.p8@dc.tohoku.ac.jp (M.T.); k.mika@med.tohoku.ac.jp (M.K.K.); 2 Department of Antibody Drug Development, Tohoku University Graduate School of Medicine, 2-1 Seiryo-machi, Aoba-ku, Sendai 980-8575, Japan

**Keywords:** mouse CCR6, monoclonal antibody, epitope, ELISA, SPR

## Abstract

CC chemokine receptor 6 (CCR6) is one of the members of the G-protein-coupled receptor (GPCR) family that is upregulated in many immune-related cells, such as B lymphocytes, effector and memory T cells, regulatory T cells, and immature dendritic cells. The coordination between CCR6 and its ligand CC motif chemokine ligand 20 (CCL20) is deeply involved in the pathogenesis of various diseases, such as cancer, psoriasis, and autoimmune diseases. Thus, CCR6 is an attractive target for therapy and is being investigated as a diagnostic marker for various diseases. In a previous study, we developed an anti-mouse CCR6 (mCCR6) monoclonal antibody (mAb), C_6_Mab-13 (rat IgG_1_, kappa), that was applicable for flow cytometry by immunizing a rat with the N-terminal peptide of mCCR6. In this study, we investigated the binding epitope of C_6_Mab-13 using an enzyme-linked immunosorbent assay (ELISA) and the surface plasmon resonance (SPR) method, which were conducted with respect to the synthesized point-mutated-peptides within the 1–20 amino acid region of mCCR6. In the ELISA results, C_6_Mab-13 lost its ability to react to the alanine-substituted peptide of mCCR6 at Asp11, thereby identifying Asp11 as the epitope of C_6_Mab-13. In our SPR analysis, the dissociation constants (*K*_D_) could not be calculated for the G9A and D11A mutants due to the lack of binding. The SPR analysis demonstrated that the C_6_Mab-13 epitope comprises Gly9 and Asp11. Taken together, the key binding epitope of C_6_Mab-13 was determined to be located around Asp11 on mCCR6. Based on the epitope information, C_6_Mab-13 could be useful for further functional analysis of mCCR6 in future studies.

## 1. Introduction

The CC chemokine receptor 6 (CCR6) is a seven-transmembrane chemokine receptor belonging to the G-protein-coupled receptor (GPCR) family [1,2,3,4,5]. CCR6 was identified as a specific receptor for the CC motif chemokine ligand 20 (CCL20) in 1997 [6]. It is reportedly associated with various diseases, such as cancer [7,8,9], autoimmune diseases [10,11,12,13], psoriasis [14,15,16,17], and inflammatory bowel disease (IBD) [18,19,20,21,22]. The expression of CCR6 is found in B cells or T cells [23], such as effector memory T cells [24], immature dendritic cells [25], Th17 cells [26], and regulatory T (Treg) cells [27], and thus affects the activity and directionality of immune cells [23,24,26]. Mice lacking CCR6 exhibited impaired leukocyte homeostasis, which results in severe contact hypersensitivity and defects in delayed-type hypersensitivity responses. These results suggested that CCR6 plays a critical role in the regulation of leukocyte homeostasis [28].

The chemokine ligand CCL20, also known as macrophage inflammatory protein-3α (MIP-3α) [29], liver- and activation-regulated chemokine (LARC) [30], or Exodus-1 [31], is a crucial CCR6 ligand. The binding of CCL20 to CCR6 can activate a variety of intracellular signaling pathways, including the calcium signaling, PI3K-Akt, MEK-ERK, STAT3, and NF-κB pathways [32]. These pathways play essential roles in the differentiation, migration, and plasticity of CD4^+^ T lymphcytes [33,34,35,36]. These findings suggest that CCR6-CCL20 signaling could provoke cross-talk with the signalling of T-cell receptors and cytokines to regulate the mitigation of CD4^+^ T lymphcytes in the inflammatory microenvironment.

CCL20 is secreted by various immune-related cells, such as B cells [23], Th17 cells [37], dendritic cells [38], and natural killer cells [39]. Although various CC chemokine receptor–ligand pairs exist, the CCR6/CCL20-regulated immune response has currently become a focus of immunological research with respect to disease development [10,38,40,41]. The expression of CCR6 and CCL20 has been found to be dysregulated in the colonic mucosa and serum from IBD patients [20,22]. CCR6^+^ T lymphcytes are involved in an imiquimod-induced psoriasis model [42]. Furthermore, the tumor-promoting effects of CCR6/CCL20 within the tumor microenvironment have been reported in many cancer types, such as renal cell carcinoma [43], gastric cancer [44], cervical cancer [45], and lung cancer [46,47]. Treg cells in peripheral blood (~60%) express CCR6, presenting increased suppressive activity and higher FOXP3 expression in patients with oral squamous cell carcinoma [48]. These findings have made the CCL20/CCR6 axis an attractive therapeutic target for various diseases., and inhibitors targeting the CCR6/CCL20 axis are also being actively developed [10].

Previously, we developed various monoclonal antibodies (mAbs) against chemokine receptors, including mouse CCR2 [49], mouse CCR3 [50], mouse CCR4 [51], mouse CCR6 (mCCR6) [52], mouse CCR9 [53], and mouse CXCR6 [54]. The N-terminus of GPCRs, including CCR6, CCR9, and CXCR6, has been identified as a ligand-binding domain [55,56,57,58]. Interestingly, the binding between CCL20 and CCR6 has been elucidated [59]. CCR6 and CCL20 have shallow binding modes on the receptor surface, which induce allosteric conformational changes and are considered to trigger binding to intracellular G-proteins [59]. Analysis of the ligand-binding mode and the characterization of antibody epitopes are important for predicting neutralizing activity and assessing efficacy against antigens.

In this study, we performed an epitope identification of a rat anti-mCCR6 mAb (C_6_Mab-13; IgG_1_, kappa) using enzyme-linked immunosorbent assay (ELISA) and surface plasmon resonance (SPR) analysis against the alanine-substituted N-terminal peptides of mCCR6.

## 2. Materials and Methods

### 2.1. Antibodies

The rat anti-mCCR6 mAb (clone C_6_Mab-13) used herein was previously developed [52]. In summary, one rat was intraperitoneally immunized with a keyhole-limpet-hemocyanin (KLH)-conjugated N-terminal peptide of mCCR6 (1–19 amino acids (aa) + C-terminal cysteine). Subsequently, the hybridoma supernatants were screened with the mCCR6p1-19C peptide using ELISA following flow cytometry using mCCR6-overexpressed CHO-K1 cells and endogenously mCCR6-expressed P388 (mouse lymphoid neoplasma) and J774-1 (mouse macrophage-like) cells [52].

We purchased secondary peroxidase-conjugated anti-rat immunoglobulins from Sigma-Aldrich Corp. (St. Louis, MO, USA).

### 2.2. Peptides

The mCCR6 (Accession No.: NM_001190333.1) peptide (_1_-MNSTESYFGTDDYDNTEYYS-_20_) and 1× alanine residue-substituted peptides (Table 1) were synthesized utilizing PEPscreen (Sigma-Aldrich Corp.).

### 2.3. ELISA

Synthesized mCCR6 peptides were immobilized on Nunc Maxisorp 96-well immunoplates (Thermo Fisher Scientific Inc., Waltham, MA, USA) at 10 µg/mL for 30 min at 37 °C. After being washed with phosphate-buffered saline (PBS) containing 0.05% Tween20 (PBST; Nacalai Tesque, Inc., Kyoto, Japan), the wells were blocked with 1% bovine serum albumin (BSA)-containing PBST for 30 min at 37 °C. The plates were incubated with 10 µg/mL of C_6_Mab-13 for 30 min at 37 °C followed by peroxidase-conjugated anti-rat immunoglobulins (1:20,000 diluted; Sigma-Aldrich Corp.) for 30 min at 37 °C. Enzymatic reactions were conducted at room temperature using the ELISA POD Substrate TMB Kit (Nacalai Tesque, Inc.). Optical density was measured at 655 nm using an iMark microplate reader (Bio-Rad Laboratories, Inc., Berkeley, CA, USA).

### 2.4. Measurement of Dissociation Constants Using Surface Plasmon Resonance (SPR)

The dissociation constants (*K*_D_) between C_6_Mab-13 and the epitope region peptides were measured using SPR. C_6_Mab-13 was immobilized on the CM5 sensor chip according to the manufacturer’s protocol (Cytiva, Marlborough, MA, USA). In summary, C_6_Mab-13 was diluted to 10 μg/mL by an acetate buffer (pH 4.0; Cytiva) and immobilized using an amine-coupling reaction. The surface of flow cell 2 of the CM5 sensor chip was treated with 1-ethyl-3-(3-dimethylaminopropyl)-carbodiimide and *N*-hydroxysuccinimide (NHS), followed by an injection of C_6_Mab-13. The unreacted NHS-ester was blocked with ethanolamine after C_6_Mab-13 immobilization. The *K*_D_ between C_6_Mab-13 and mCCR6 peptides (50, 25, 12.5, 6.25, and 3.13 µM) were measured using Biacore X100 (Cytiva) at 25 °C. The buffer was filtrated with PBS containing 0.05% (*v*/*v*) Tween 20 and 0.24% (*v*/*v*) dimethyl sulfoxide (FUJIFILM Wako Pure Chemical Corporation, Osaka, Japan). The single-cycle kinetics method was used to measure the binding signals. The data were analyzed using 1:1 binding kinetics to determine the association rate constant (*k*_a_), dissociation rate constant (*k*_d_), and *K*_D_ using BIAevaluation software (Cytiva).

## 3. Results

### 3.1. Epitope Identification of C_6_Mab-13 by ELISA Using 1× Alanine-Substituted mCCR6 Peptides

**Figure 1:**
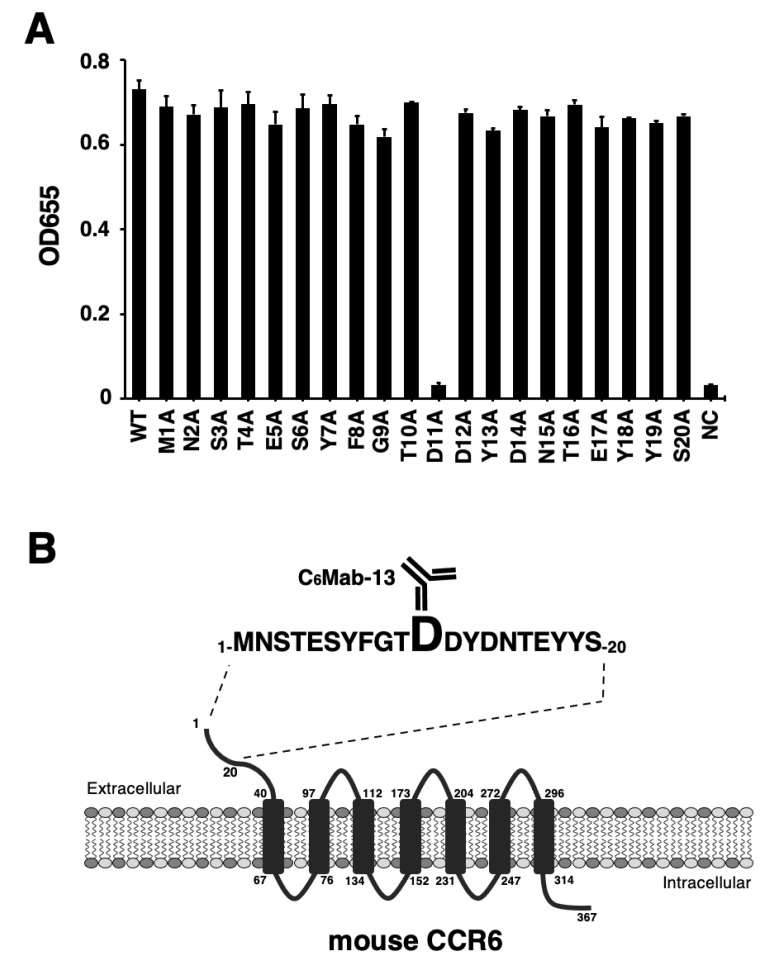
Determination of the C_6_Mab-13 epitope by ELISA using alanine-substituted peptides of mCCR6. (**A**) Synthesized peptides of mCCR6 (10 µg/mL) were immobilized on immunoplates for 30 min at 37 °C. The plates were incubated with 10 µg/mL of C_6_Mab-13, followed by treatment with peroxidase-conjugated anti-rat immunoglobulins. Optical density was measured at 655 nm (OD655) using a microplate reader. (**B**) The schematic illustration of mCCR6 and the C_6_Mab-13 epitope. The C_6_Mab-13 epitope of mCCR6 comprises Asp11 from ELISA experiments.

We previously developed an anti-mCCR6 mAb, C_6_Mab-13 (rat IgG_1_, kappa), by immunizing a rat with a KLH-conjugated mCCR6 N-terminal peptide [52]. C_6_Mab-13 is applicable to ELISA and is useful for detecting mCCR6-expressing cells via flow cytometry [52]. To characterize the binding epitope of C_6_Mab-13, we synthesized 20 different 1× alanine-substituted mCCR6 peptides between Met1 to Ser20. The sequences are listed in Table 1. The results of ELISA using alanine-substituted peptides and C_6_Mab-13 demonstrated that C_6_Mab-13 bound to point mutants, such as M1A, N2A, S3A, T4A, E5A, S6A, Y7A, F8A, G9A, T10A, D12A, Y13A, D14A, N15A, T16A, E17A, Y18A, Y19A, and S20A, as well as the 1–20 aa wild-type (WT) sequence (positive control) (Figure 1A). In contrast, C_6_Mab-13 did not react with the D11A peptide (Figure 1A). These results indicate that Asp11 was the critical aa, which is included in the C_6_Mab-13 epitope. The results are summarized schematically in Figure 1B.

### 3.2. Epitope Identification of C_6_Mab-13 by SPR Using 1× Alanine-Substituted mCCR6 Peptides

To confirm the C_6_Mab-13 epitope, we measured the binding affinity between C_6_Mab-13 and the synthesized peptides, including 20 point mutants and the WT of mCCR6, using Biacore X100. The peptides’ sequences are presented in Table 1, and the measured values are summarized in Table 2. The k_a_, k_d_, and *K*_D_ of G9A and D11A were not determined. These results demonstrated that Gly9 and Asp11 were the critical amino acids of the C_6_Mab-13 epitope.

Mutant peptides of F8A, T10A, Y13A, and D14A increased the *K*_D_ values by 15.5-, 4.4-, 16.5-, and 2.8-fold, respectively (Table 2), indicating that Phe8, Thr10, Tyr13, and Asp14 may contribute to the binding of C_6_Mab-13 to mCCR6.

## 4. Discussion

This study examined the binding epitope of C_6_Mab-13 through a 1× alanine-substituted-peptide-scanning method using ELISA and SPR. We concluded that Asp11 is a pivotal epitope aa in ELISA, while Gly9 and Asp11 are critical in SPR. This epitope is located outside the region of all three extracellular domains of CCR6 and N-terminal residues from Tyr27 to Leu38, to which the chemokine ligand CCL20 binds [59,60]. There is a possibility that structural changes might occur upon C_6_Mab-13′s binding to CCR6, which leads to allosteric effects on CCL20 binding. Therefore, we will investigate the neutralizing activity of C_6_Mab-13 between CCL20 and CCR6 in the future study.

A recent report showed that low rather than high affinity of mAb to a target provokes elevated activity through inducing the clustering of receptors. These findings provide new insights for antibody-mediated receptor signaling [61]. Since CCR6 is involved in intracellular signaling [62], the relationship between antibody affinity and the effect of cellular signaling should be investigated in future studies.

The epitope-mapping results obtained using ELISA (Figure 1) and SPR (Table 2) indicated a similar region of mCCR6 as the binding epitope. However, Gly9 was only identified as the critical aa by via SPR analysis (Table 2). The experimental system differs between both experiments, as follows: (i) for ELISA, the synthesized peptides were immobilized on immunoplates, while C_6_Mab-13 was immobilized on a CM5 sensor chip in the SPR analysis; (ii) the reaction times between the antigen and the antibody were different; and (iii) the secondary antibody was only used for ELISA. These different conditions may have precipitated the inconsistent results of both experiments in this study.

In the SPR analysis, mutant peptides of F8A, T10A, Y13A, and D14A increased the *K*_D_ values by 15.5-, 4.4-, 16.5-, and 2.8-fold, respectively (Table 2). These results indicate that Phe8, Thr10, Tyr13, and Asp14 may contribute to C_6_Mab-13′s binding to mCCR6. In the future, we will adopt the cell-based alanine- or 2× alanine-scanning methods for a detailed epitope analysis of C_6_Mab-13, as we have clarified the epitope of mAb [63].

When CCL20 is secreted in tumor tissues [64], it attracts CCR6-expressing Treg cells [65], which are involved in tumor progression and poor prognosis [66,67]. Therefore, novel cancer treatment strategies using CCR6-expressing chimeric antigen receptor-T (CAR-T) cells have been designed [68,69]. Furthermore, removing immunosuppressive cells, such as CCR6+ Treg cells, may enhance antitumor efficacy [70]. In this study, we demonstrated that C_6_Mab-13 possesses high binding affinity against mCCR6, which was expressed in Chinese hamster ovary-K1 cells (*K*_D_: 2.8 × 10^−9^ M according to flow cytometric analysis) [52]. Therefore, C_6_Mab-13 is expected to be useful for antitumor evaluations considering the depletion of CCR6-expressing Treg cells in mouse models.

## Figures and Tables

**Table 1:** Identification of C_6_Mab-13 epitope using point mutant peptides of mCCR6 via enzyme-linked immunosorbent assay.

Peptides	Sequences	C_6_Mab-13 Reactivity
p1–20 (WT)	MNSTESYFGTDDYDNTEYYS	+++
M1A	ANSTESYFGTDDYDNTEYYS	+++
N2A	MASTESYFGTDDYDNTEYYS	+++
S3A	MNATESYFGTDDYDNTEYYS	+++
T4A	MNSAESYFGTDDYDNTEYYS	+++
E5A	MNSTASYFGTDDYDNTEYYS	+++
S6A	MNSTEAYFGTDDYDNTEYYS	+++
Y7A	MNSTESAFGTDDYDNTEYYS	+++
F8A	MNSTESYAGTDDYDNTEYYS	+++
G9A	MNSTESYFATDDYDNTEYYS	+++
T10A	MNSTESYFGADDYDNTEYYS	+++
D11A	MNSTESYFGTADYDNTEYYS	-
D12A	MNSTESYFGTDAYDNTEYYS	+++
Y13A	MNSTESYFGTDDADNTEYYS	+++
D14A	MNSTESYFGTDDYANTEYYS	+++
N15A	MNSTESYFGTDDYDATEYYS	+++
T16A	MNSTESYFGTDDYDNAEYYS	+++
E17A	MNSTESYFGTDDYDNTAYYS	+++
Y18A	MNSTESYFGTDDYDNTEAYS	+++
Y19A	MNSTESYFGTDDYDNTEYAS	+++
S20A	MNSTESYFGTDDYDNTEYYA	+++

+++, OD655 ≧ 0.3; -, OD655 < 0.1.

**Table 2:** The *K*_D_ between C_6_Mab-13 and 1× alanine-substituted peptides determined by surface plasmon resonance.

Peptides	*k*_a_ (/ms)	*k*_d_ (/s)	*K*_D_ (M)
p1_20 (WT)	6.84 × 10^3^	3.77 × 10^−3^	5.52 × 10^−7^
M1A	6.94 × 10^3^	4.15 × 10^−3^	5.99 × 10^−7^
N2A	7.86 × 10^3^	4.23 × 10^−3^	5.38 × 10^−7^
S3A	7.62 × 10^3^	4.53 × 10^−3^	5.94 × 10^−7^
T4A	7.92 × 10^3^	4.55 × 10^−3^	5.75 × 10^−7^
E5A	8.20 × 10^3^	4.64 × 10^−3^	5.65 × 10^−7^
S6A	9.05 × 10^3^	5.25 × 10^−3^	5.81 × 10^−7^
Y7A	8.16 × 10^3^	3.45 × 10^−3^	4.23 × 10^−7^
F8A	1.43 × 10^3^	1.23 × 10^−2^	8.55 × 10^−6^
G9A	ND	ND	ND
T10A	1.31 × 10^4^	3.15 × 10^−2^	2.40 × 10^−6^
D11A	ND	ND	ND
D12A	7.43 × 10^3^	7.09 × 10^−3^	9.55 × 10^−7^
Y13A	1.43 × 10^3^	1.30 × 10^−2^	9.12 × 10^−6^
D14A	6.87 × 10^3^	1.05 × 10^−2^	1.53 × 10^−6^
N15A	6.19 × 10^3^	5.61 × 10^−3^	9.06 × 10^−7^
T16A	6.23 × 10^3^	5.17 × 10^−3^	8.30 × 10^−7^
E17A	6.38 × 10^3^	6.67 × 10^−3^	1.05 × 10^−6^
Y18A	5.23 × 10^3^	5.56 × 10^−3^	1.06 × 10^−6^
Y19A	5.75 × 10^3^	6.02 × 10^−3^	1.05 × 10^−6^
S20A	4.68 × 10^3^	5.96 × 10^−3^	1.27 × 10^−6^

WT, wild-type; ND, not determined.

## Data Availability

All related data and methods are presented in this paper. Additional inquiries should be addressed to the corresponding authors.
